# Supplementary material for: Spatiotemporal dynamics of land use land cover change and its drivers in the western part of Lake Abaya, Ethiopia
Source: PeerJ. 2024 Sep 18;12:e17892. doi: 10.7717/peerj.17892 (PMC11416075; doi:10.7717/peerj.17892)
Supplement: Supplemental Information 1 [file peerj-12-17892-s001.docx]

Table S1

| **Driver** | **%** | **Rank** |
| --- | --- | --- |
| Agricultural land expansion | 45% | 1 |
| Settlement expansion | 22% | 2 |
| Charcoal production | 12% | 3 |
| Firewood collection | 10% | 4 |
| Tree cutting for other purposes | 7% | 5 |
| Overgrazing | 4% | 6 |
| Total | 100% |  |
