# Supplementary material for: Spatiotemporal dynamics of land use land cover change and its drivers in the western part of Lake Abaya, Ethiopia
Source: PeerJ. 2024 Sep 18;12:e17892. doi: 10.7717/peerj.17892 (PMC11416075; doi:10.7717/peerj.17892)
Supplement: Supplemental Information 2 [file peerj-12-17892-s002.docx]

Table S2

| **Driver** | **%** | **Rank** |
| --- | --- | --- |
| Population pressure | 55 | 1 |
| Poverty and unemployment | 32 | 2 |
| Climate change (precipitation variability) | 8.4 | 3 |
| Policy and institutional factors | 4.6 | 4 |
| Total | 100 |  |
