# Supplementary material for: Spatiotemporal dynamics of land use land cover change and its drivers in the western part of Lake Abaya, Ethiopia
Source: PeerJ. 2024 Sep 18;12:e17892. doi: 10.7717/peerj.17892 (PMC11416075; doi:10.7717/peerj.17892)
Supplement: Supplemental Information 6 [file peerj-12-17892-s006.docx]

Checklist of semi-structured interview question for **LULC change data collection**

**I. Key informant interview guide**

1. **General Information:**
   - Date: _____________________
   - Name of the respondent: _____________________ Gender: M__ F___ Age: ____
   - Marital status: Married____ widowed____ divorced____ single____
   - Religion: Orthodox___ Muslim____ Protestant____ Other_____
   - Educational status: Illiterate/Literate-Read and Write? (Yes___ grade___; No_ _)
2. How long did you live in the area? ______.
3. How is the land history of your district?
4. How the land usage was looks like in your areas over the past 30 years?
5. Do you think that the land is becoming scarce or it is still abundant in your community?
   1. If your answer for question no. 4 is becoming scarce, what are your reasons?
   2. If your answer for question no. 4 is still abundant, what are your justifications?
6. Have you noticed any change in the LU/LC in your area over the past 30 years?
7. Is there any change observed in your area with regard to vegetation cover, settlement areas, cultivation land and land use pattern over the past 30 years?
8. Did the changes also modify the land cover types in any ways?
9. What are the driving forces of land use/land cover changes in your area?
   1. Lack of proper management
   2. population growth
   3. Increasing demand for farming Technology
   4. Settlement expansion
   5. To expand farming land
   6. Other (specify):
10. Has the quality of the forest and crop land changed over the last 30 years?
11. Do you have an awareness of proper land use and management? Please, discuss it.
12. What are the new skills that you obtained from the district rural land management experts?
13. What are effects of land use/land cover change in the areas?
14. Soil erosion /land degradation
15. Deforestation
16. Decreasing of crop yields.
17. Migration and extinction of wild animals.
18. Climate change
19. Other (specify):
20. What are your recommendations to conserve these resources and manage these land use/land cover change?

**II. Group discussion checklist**

1. General information for FGD participants
   - Date _____________________
   - Name of the respondent _____________________ Gender: M__ F__ _ Age: ____
   - Marital status: Married____ widowed____ divorced____ single____
   - Religion: Orthodox___ Muslim____ Protestant____ Other____ _
   - What is your level of education?
   - What is your qualification?
   - Your work experiences?
   - What is your role of expert in the office?
2. Is there any change of land use/land cover over time and space in your district and what are the major driving forces behind?
3. Is there any investment or investor on land improvement measures? Yes/No, if yes, what type of improvement measures exist?
4. Can you mention the consequences of land cover changes from one type to another?
5. How local people’s occupation of land changed over the past 30 years, because of population increment?
6. Do the individuals or the community actively participating in the conservation activities?
7. Do you believe that human activities have an immediate impact on land use land cover change?
8. What are effects of land use/land cover change in the areas?
9. What new skills you thought the rural population in relation to land use management?
10. Do you think that the rural land management and environmental protection sector has helped the rural population to conserve their own natural resources?
11. What are your recommendations to conserve these resources and manage these land use/land cover change medicinal plant species?
